# The Microenvironment in Immobilized Enzymes: Methods of Characterization and Its Role in Determining Enzyme Performance

**DOI:** 10.3390/molecules24193460

**Published:** 2019-09-24

**Authors:** Juan M. Bolivar, Bernd Nidetzky

**Affiliations:** 1 Institute of Biotechnology and Biochemical Engineering, Graz University of Technology, NAWI Graz, Petersgasse 12, A-8010 Graz, Austria; 2 Chemical and Materials Engineering Department, Complutense University of Madrid, 28040 Madrid, Spain; 3 Austrian Centre of Industrial Biotechnology (acib), Petersgasse 14, A-8010 Graz, Austria

**Keywords:** biocatalysis, immobilization, microenvironment, internal milieu, reaction-diffusion, opto-chemical sensing, pH, oxygen, porous materials

## Abstract

The liquid milieu in which enzymes operate when they are immobilized in solid materials can be quite different from the milieu in bulk solution. Important differences are in the substrate and product concentration but also in pH and ionic strength. The internal milieu for immobilized enzymes is affected by the chemical properties of the solid material and by the interplay of reaction and diffusion. Enzyme performance is influenced by the internal milieu in terms of catalytic rate (“activity”) and stability. Elucidation, through direct measurement of differences in the internal as compared to the bulk milieu is, therefore, fundamentally important in the mechanistic characterization of immobilized enzymes. The deepened understanding thus acquired is critical for the rational development of immobilized enzyme preparations with optimized properties. Herein we review approaches by opto-chemical sensing to determine the internal milieu of enzymes immobilized in porous particles. We describe analytical principles applied to immobilized enzymes and focus on the determination of pH and the O_2_ concentration. We show measurements of pH and [O_2_] with spatiotemporal resolution, using in operando analysis for immobilized preparations of industrially important enzymes. The effect of concentration gradients between solid particle and liquid bulk on enzyme performance is made evident and quantified. Besides its use in enzyme characterization, the method can be applied to the development of process control strategies.

## 1. Introduction

Enzymes are powerful catalysts of chemical reactions of synthetic importance [1,2,3,4]. The practical use of enzymes in diverse applications often involves a solid catalyst preparation [1,5,6]. The solid catalyst is obtained through enzyme immobilization [5,6]. Immobilization entails enzyme attachment to, or encapsulation in, a suited solid material. Different principles of enzyme immobilization have been described in almost countless varieties [5,7]. However, the most common strategy of enzyme immobilization utilizes a mesoporous solid support as the enzyme carrier [5,6,8]. The support is selected to offer a high internal surface area accessible to the enzyme [5,8,9,10]. The surface is chemically suitable for the enzyme to become attached physically, chemically or both [5,7]. Thus immobilized enzymes are widely used in heterogeneous biocatalyses applied to organic synthesis, up to the industrial manufacturing scale [1,2,3,4].

**Figure 1:**
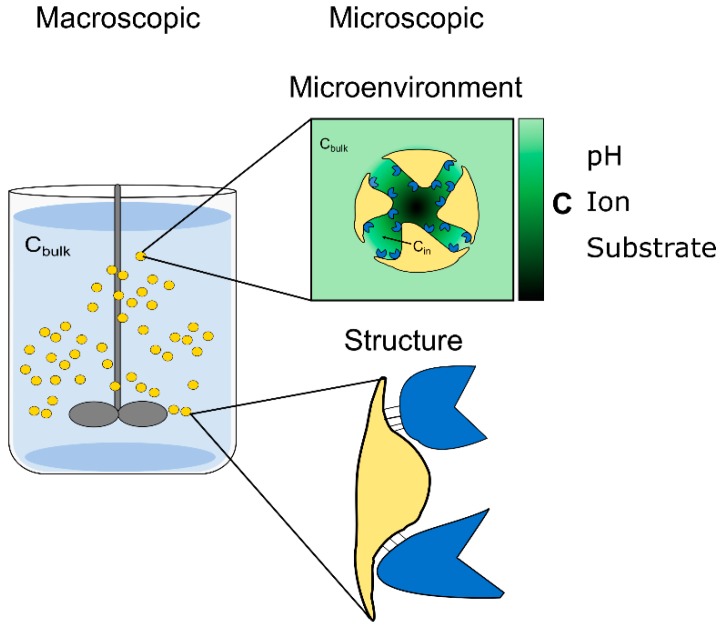
Factors influencing the macroscopic behavior of immobilized enzymes. The formation of a distinct microenvironment in porous enzyme supports is shown. The analyte’s concentration in the well-mixed liquid bulk (C_bulk_) often differs from the concentration inside the carrier, referred to as the internal milieu (C_in_). Spatially resolved measurements are needed to obtain the full internal profile of C_in_, represented by the light-to-dark green scale. Enzyme binding to the solid surface potentially alters the protein structure. The figure was adapted from reference [14] with permission from Elsevier.

An enzyme immobilized within a porous solid support is sequestered from the surrounding bulk solution. Catalysis happens under the confinement of the solid surface to which the enzyme is attached [11]. Therefore, immobilized enzymes commonly differ in their apparent properties (i.e., activity and stability) from those of the soluble counterparts [6,12]. The mechanistic challenge for the study of immobilized enzymes is to characterize effects on an enzyme’s properties due to the particular microenvironment in the solid material [12,13,14]. We are concerned primarily with the effect of sequestration, affecting the liquid milieu in which the immobilized enzyme operates. This “intraparticle” liquid milieu can be quite different from the milieu in bulk solution [12,14] (Figure 1). These differences may be in the substrate or product concentration, but can also be in pH, cosolvent concentration and ionic strength [12,14]. The effects of enzyme binding to the solid surface are also highly important [6,15,16,17]. These latter effects are considered herein, only insofar as their quantitative assessment, unmasked from the effects of the milieu, is concerned. The suitable detection of structural distortions relevant for enzyme function, ideally at single-molecule resolution, remains a significant challenge for immobilized enzymes [12,17,18,19,20].

The internal milieu for immobilized enzymes is affected by two factors in particular. Firstly, there is solute partitioning between the liquid phase and the solid phase of the carrier. The partitioning is due to the chemical properties of the solid material (e.g., hydrophobicity and charge), creating a distinct surface-near environment with respect to polarity, dielectric and ionic strength [5,6,21,22]. Secondly, there is the interplay between reaction and diffusion. Mass transfer by diffusion occurs from the liquid bulk to the surface of the carrier and from the surface into the pores of the particle. When diffusion is not much faster than reaction, gradients in the substrate and product concentration arise between the bulk and the carrier. At steady state, therefore, the chemical milieu in bulk and in particle is different [12,14]. Enzyme performance is influenced by the changes in microenvironment (Figure 1). A key performance parameter is the catalytic rate (activity). This depends on substrate and product concentration, as well as on pH and ionic strength. Besides the kinetic effect, changes in the critical concentrations can affect the reaction equilibrium constant and the effective mass-action ratio in the solid particle. Another important enzyme parameter is stability. This may also be affected by changes in milieu parameters; the pH, for example.

It follows from the above that the characterization of the microenvironment of an immobilized enzyme is essential for the understanding of the overall performance of the catalyst [12,13,20]. Kinetic theory accounts for the effect of coupled reactions and diffusion and is a useful predictive tool [23]. However, unless critical reaction parameters are measured directly from the porous support, experimental verification of the kinetic theory is limited. However, kinetic parameters are normally determined exclusively from the convoluted data recorded in liquid bulk. Experimental methods for the evaluation of intrinsic kinetic parameters under the existence of partition effects and mass transfer resistances are described in excellent reviews and textbooks [23,24]. The study of how apparent kinetic parameters of the enzyme (e.g., K_m_) change in their dependence on variables of the immobilization process (e.g., material chemistry and geometrical features of the solid support) can be useful [6,9,25]. However, the apparent kinetic parameters provide indirect evidence of a convoluted nature. Therefore, determination of differences in the internal compared to the bulk milieu is fundamentally important in the mechanistic characterization of immobilized enzymes [12,14]. The deepened understanding thusly obtained is critical for the rational development of immobilized enzyme preparations with optimized catalytic properties.

Our main goal here is to review correlations between the internal environment and the functional behavior of enzymes immobilized in solid materials. We discuss approaches by opto-chemical sensing to determine the composition of that internal environment. We describe the relevant analytical principles applied to the study of immobilized enzymes. Although opto-chemical sensing has been developed for a wide variety of solutes, we focus herein in particular on the determination of the pH and of the O_2_ concentration. We show measurements of internal pH and [O_2_] with spatiotemporal resolution, using in operando analysis for immobilized preparations of industrially important enzymes. We provide experimental evidence for the development of substantial concentration gradients between the solid particle and the liquid bulk. These gradients are quantified and their effect on enzyme performance is determined. We show that, besides its use for characterization of solid-supported immobilized enzymes, the method of optical sensing in solid materials can be useful for the development of process control strategies.

## 2. Opto-Chemical Sensing within Solid Particles

### 2.1. The Principles of Opto-Chemical Sensing

**Figure 2:**
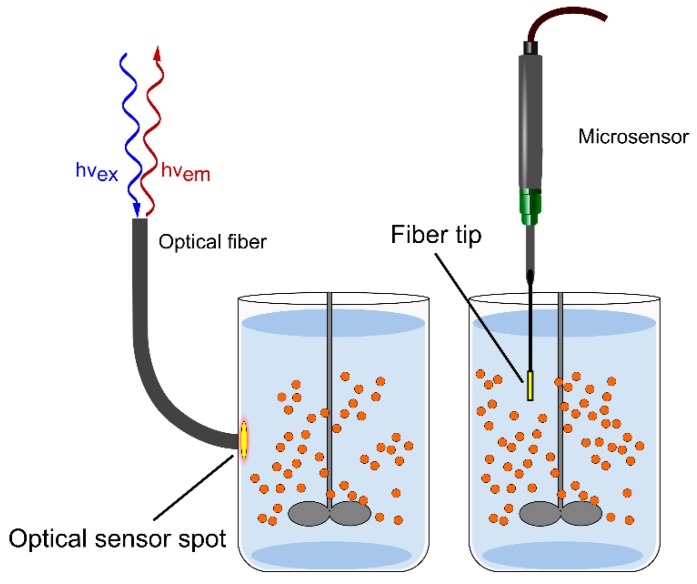
Optical sensing in solution. An integrated sensor spot (left) or a microsensor (middle) can be used for the determination of pH, O_2_ and other analytes. The figure was adapted from reference [14] with permission from Elsevier.

Various analytical techniques are available for the characterization of the internal microenvironment in solid-supported catalysts (e.g., NMR, Raman and IR) [12,20,26,27,28,29,30,31,32]. Among these techniques, opto-chemical sensing offers the specific advantage that both the external (Figure 2) and the overall (space-averaged) internal concentrations of the analyte of interest can be measured at the same time, using a single analytical device [12,14]. Opto-chemical sensors have gained considerable importance in the analytical sciences. Principles of methodology and technologies for measurement are well established [33,34]. Sensors for O_2_ are widely applied in the environmental sciences, in (bio)process engineering and in the life sciences. Based on experience with the opto-chemical sensing of oxygen, strategies for measuring other analytes (e.g., pH) have been developed. Principles have been extended to the determination of CO_2_, NH_4_, glucose, alcohols, amines and a variety of ions [33,35]. Temperature can be also measured [36]. Self-fluorescent molecules (e.g., NADH and NADPH) might lend themselves directly for (internal) sensing using fluorescence resonance energy transfer [37,38,39]. In this section, the principles of opto-chemical sensing are reviewed. Their implementation in analytical strategies for the characterization of the intraparticle microenvironment in immobilized enzymes is discussed.

For sensing in liquid solution, opto-chemical sensors are commercially available in numerous formats, such as layers or spots, fiber-optic (micro)sensors, and sensor particles in micro or nanometer sizes [40,41,42,43,44] (Figure 2). The basic format consists of indicator molecules immobilized in an analyte-permeable polymer layer. The sensitive material is further system-integrated by controlled deposition as in sensor spots and optical fiber tips, enabling different and often contactless optical readouts; e.g., from the wall of a transparent reaction vessel [45] (Figure 2). The sensor format determines the mode, or state, of sensor integration into the enzyme reactor [46]. The different formats allow for a non-invasive flexible application at suitable measurement positions, and for an analytic read-out at the microscopic scale [46,47]. The sensors can be employed as single analyte detection units or as multi-parameter detection units. Quantification of diverse analytes in the homogeneous liquid phase, or in proximity to a solid-liquid interface, is, therefore, possible with ready-to-use technologies. The method is flexibly applicable to very different reactor configurations, from reaction vessels to microfluidic systems working as continuous flow reactors [34,45,46]. Moreover, opto-chemical sensing can be applied largely independent of geometry, scale and operational parameters of the reactor used.

**Figure 3:**
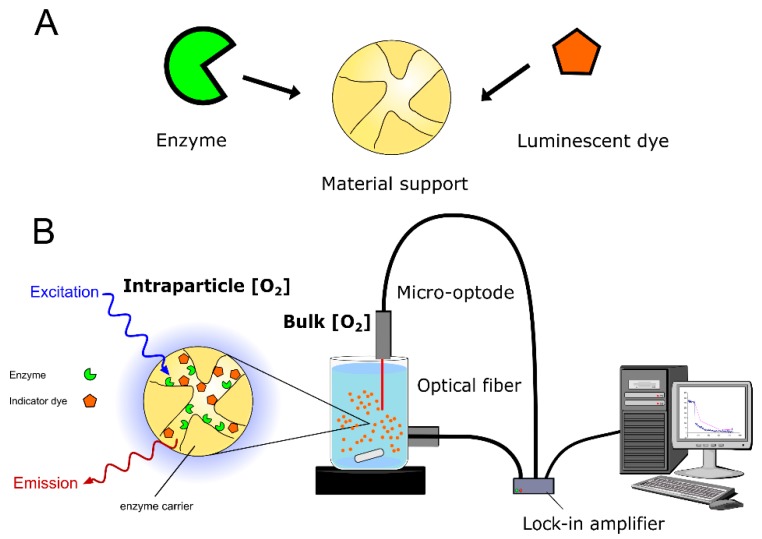
Opto-chemical sensing in enzyme immobilizates. (**A**) Preparation of analyte-sensitive carrier material and enzyme immobilization. (**B**) Application to space-averaged, time-resolved determination of internal concentrations. Figure 3B was adapted from reference [48] with permission from John Wiley and Sons.

The features just discussed make opto-chemical sensing to be particularly apt for being used in heterogeneous (solid-liquid) environments. However, its application for analysis inside solid porous supports has two additional, specific requirements. First, the luminescence dye and the enzyme should be properly co-immobilized within the same material; that way, the solid support would be internally sensitive to the analyte to be detected (Figure 3). Second, a set-up for read-out should be established that provides measurements with suitable spatiotemporal resolutions (Figure 3). As a general principle, the labeling of the solid material needs to be compatible with the procedure of enzyme immobilization. Choice of the analytical set-up determines which reactor configuration is suitable for luminescence measurement [14,48]. It also determines which level of spatial resolution can be obtained. We discuss method development using recent examples from the literature.

### 2.2. Luminescence Labeling of the Solid Support

The procedure of labeling with the luminescence dye used must ensure that the properties of the immobilized enzyme are minimally affected. It should also be compatible with the methods of preparation of the immobilized enzyme, or require minimal modifications in them. Additionally, the labeling procedure must ensure that the labeled material offers a suitable analytical response. One possibility for luminescence labeling is the direct conjugation of the enzymes with the luminescence dye [49,50,51]. This direct conjugation can be performed before or after the immobilization of the enzyme. Working with a labeled enzyme, however, one must be aware that the procedure of immobilization could be affected. The main challenges of enzyme labeling before immobilization are twofold. First, the enzyme activity is typically changed as a consequence of the chemical conjugation. Second, the mass amount of dye incorporated into the solid is often low. This can cause problems with the analytical reading.

The alternative is to label the support material, which is, in general, the preferred approach for the preparation of internally sensitive material (Figure 3A). Diverse examples of luminescence labeling can be found in the literature: entrapment in alginate beads [52,53]; covalent incorporation into PEG microparticles [54] and membranes [55]; ionic adsorption on amine-activated silica surfaces [21]; covalent incorporation into porous silica or aluminum oxide pores [56,57]; and encapsulation in PVA hydrogel beads [58].

One quite practical approach of material labeling is based on hydrophobic adsorption of the luminescence dye into the carrier material. Fluorescein (a pH-sensitive dye) and tris (4,7-diphenyl-1,10-phenantroline) ruthenium (II) dichloride (an O_2_ sensitive dye; Ru(dpp)_3_Cl_2_) become stably adsorbed by carriers fabricated from polymethacrylate material, making their covalent attachment unnecessary [48,59]. A very strong hydrophobic adsorption is observed, which results in quasi-irreversible labeling of the carrier. Dye wash-out during incubation in the aqueous buffer is prevented thusly. The polymethacrylate carriers have become widely known for enzyme immobilization and are being marketed under different tradenames (e.g., Sepabeads, ReliZyme and others) [60,61]. It is worth pointing out, therefore, that luminescence labeling is fully compatible with a representative variety of reactive carrier surface groups (e.g., aldehyde, amine, carboxylic acid, diol and epoxide), which are already offered in the commercial carriers, or are able to be introduced easily on them through convenient derivatization [48,59,62]. An analysis of labeled particles by confocal laser scanning microscopy revealed that dye incorporation into the porous polymethacrylate material was spatially uniform (J.M. Bolivar *et al.*, unpublished).

Luminescence labeling through direct incorporation into the enzyme carrier, as demonstrated for the relatively hydrophobic polymethacrylate material, is applicable to a range of other organic polymers used in the field. Examples are Eupergit^®^ and Purolite^®^ carriers. More hydrophilic carriers require adaptation of the labeling procedure, using covalent fixation or other forms of deposition of the luminophore(s) on the surface. For example, hydrogels applied for encapsulation of enzymes and cells should be generally amenable to the luminescence labeling [54]. Transfer of the (simple non-covalent) luminescence labeling procedure to alternative carrier materials used in the field of enzyme immobilization, including silica and agarose [18,63], has been recently accomplished [64,65]. The Ru-based organometallic luminophore was adsorbed tightly onto the silica porous supports. Optimization of the surface labeling regarding homogeneous luminophore distribution was guided, and its efficacy verified, by CLSM [65]. Porous agarose beads (e.g., phenyl sepharose beads) were also labeled with the oxygen-sensitive dye Ru(dpp)_3_ [64].

When working with two industrial enzymes, namely cephalosporin C amidase and glucose oxidase, carrier labeling with fluorescein and Ru(dpp)_3_Cl_2_ did not affect the enzyme immobilization in any way [48,59,62]. While these findings indicate the useful bio-compatibility of the labeling procedure, they clearly cannot be generalized at large. It is, therefore, advisable to always check for dye effects on the activity or stability of the immobilized enzyme under examination. For example, studies were performed to minimize the decrease of activity of D-amino acid oxidase in the presence of the luminophore [66]. The sequence of immobilization and labeling was identified as important [65]. However, it should be emphasized that, unless there was a massive impairment of enzyme function by the immobilized dyes, the establishment of a preparation of sensitive materials for the measurement of internal concentration would still be very useful. It could be used to assess the role of diffusion in limiting the overall reaction rate.

One recent method of labeling is the use of a sensor protein instead of a sensor chemical dye [67]. The use of proteins enables a fully biocompatible methodology for real-time opto-chemical sensing within porous materials. A genetically encoded ratiometric pH indicator, the superfolder yellow fluorescent protein (sYFP), was used to functionalize the internal surface of enzyme carrier supports. By using controlled, tailor-made immobilization, sYFP was homogeneously distributed within carrier materials, and so enabled, via self-referenced imaging analysis, pH measurements with high accuracy and with useful spatiotemporal resolution (see later, Section 3). Unlike opto-chemical pH sensors, which often interfere with biological function, labeling with sYFP enables pH sensing without altering the immobilized enzyme’s properties in any of the materials used [67].

### 2.3. Choice of Set-Up and the Degree of Spatial and Temporal Resolution

**Figure 4:**
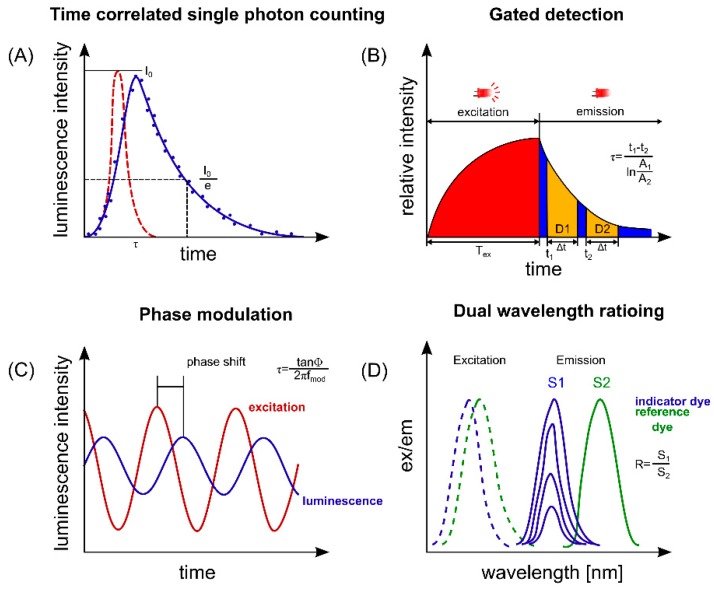
Measurement methods applied in optical sensing. (**A**) Luminescence lifetime determination by time-correlated single-photon counting. (**B**) Lifetime determination by gated detection: rapid lifetime determination is shown. (**C**) Lifetime determination by phase modulation. (**D**) Dual wavelength ratioing. The figure was reproduced from reference [14] with permission from Elsevier.

The overall set-up for measurement requires choosing the analytical principle in proper combination with an integrated read-out. Regarding the analytical principle, most of the sensors used are based on the principle of photoluminescence. The dependence of the luminescence properties on the concentration of the analyte is exploited for analytical determination. The detection involves one of the following strategies: detection of intensity, lifetime-correlated single-photon counting (TCSPC), lifetime gate detection, phase modulation technique or dual-wavelength ratioing. The strategies of measurement are illustrated in Figure 4. Their details are discussed in recent reviews [14,46]. The strategies shown in Figure 4 are commonly applied to the measurement of O_2_ and pH. Opto-chemical O_2_ sensors operate according to the principle of dynamic quenching of the phosphorescence of an indicator dye. The quenching affects both the intensity and lifetime of the phosphorescence, whereby lifetimes are typically in the range 1–100 µs. The measurement of the lifetime is generally superior compared to the measurement of intensity because it is an intrinsically referenced parameter. Contrary to intensity, it is not affected by scattering, reflection, drifts in the opto-electronic set-up, and inhomogeneous distribution or bleaching of the indicator. Lifetime can be determined in the time domain, but also in the frequency domain [35,68,69] (see Figure 4). Opto-chemical pH-sensors change their absorption or emission properties in a manner dependent of the indicator’s protonation state. Fluorescent pH-indicators exhibit lifetimes below <100 ns, requiring a higher degree of instrument sophistication compared to O_2_-sensors. However, a straightforward approach of pH measurement involves ratiometric data collection where the ratio of emission intensity at two wavelengths is determined [70]. Alternatively, a method called dual-lifetime referencing is applied to convert the intensity signal into a referenced signal, either a phase shift or a time-dependent parameter, by adding a phosphorescent reference dye to the sensing layer [71,72]. Using a slight modification of the procedure applied for single analyte determination, dual sensing of pH and O_2_ has also been reported [35].

**Figure 5:**
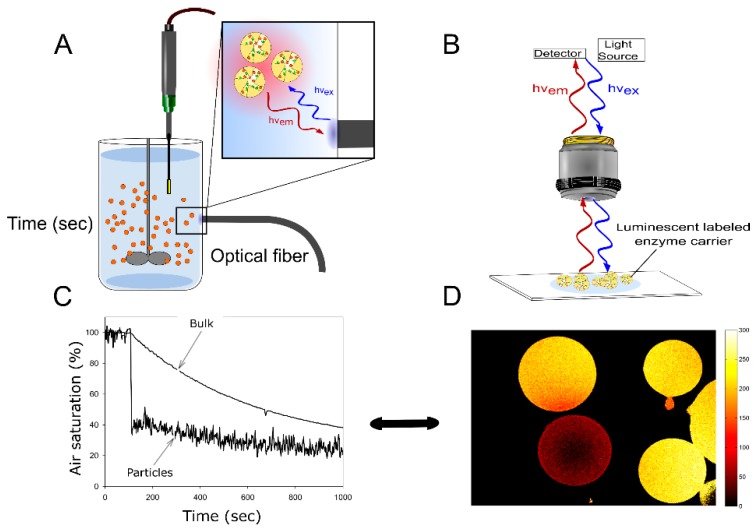
Read-out strategy and spatiotemporal resolution provided by opto-chemical sensing in enzyme immobilizates. (**A**) Interfacing fiber optics with oxygen sensitive particles for space-averaged determination of intraparticle analyte concentrations. (**B**) Interfacing opto-chemical sensing with a microscopy set-up for the spatial resolution of intraparticle concentrations in stagnant solutions or in a fixed bed. (**C**) Time courses of the average intraparticle oxygen concentration and the corresponding oxygen concentration in bulk when O_2_ is utilized as substrate by an immobilized enzyme. (**D**) Example of the spatial resolution of intraparticle oxygen concentration. The figure was adapted from reference [14] with permission from Elsevier.

Depending on the practical combination of measurement principle and sensor format, various configurations of the analytical system can be considered. Examples of often used configurations are those of fluorescence microscopes and dual optical fibers. Using optical fibers, measurements are possible for mixed suspensions of solid particles or fixed beds of particles [14,46]. Analytical set-ups for internal sensing are distinguished according to whether they provide spatial resolution of the measured parameter. The use of optical fibers offers high flexibility in that both stirred suspensions and packed beds of particles can be analyzed. The use of microscopy provides higher spatial resolution, but restricts the application to stagnant suspensions of particles or to flow-cells configurations (Figure 5) [14,46]. Figure 5 illustrates both configurations. The experimental arrangement shown in Figure 5A allows for the collection of an overall signal useful to measure globally the averaged values of the internal parameter, and it does so in real time. Data collection can be done at the same time in bulk solution and within the carrier. This enables the recording of data points in a continuous fashion and it enables the direct determination of concentration gradients between the homogeneous liquid phase and the internal milieu of the solid catalyst (Figure 5). In initial studies, the application of this general set-up involved measurements of the pH-sensitive fluorescence intensity [49]. However, fluorescence intensity measurements are disturbed by the moving particles in stirred suspension. More recently, therefore, lifetime measurements (dual-lifetime referencing method) have improved the pH resolution and have expanded the applicability of the technique [58,59,62]. Self-referenced measurements and fluorescence lifetime determinations exhibit superior analytical performance in agitated systems. Dual lifetime referencing (DLR) in particular offers high versatility, for it is independent of the catalyst’s concentration, the reactor configuration and the scale of operation. The implementation of the space-averaged measurements internal O_2_ concentration has been accomplished using the phase modulation technique. The measurement was done by interfacing a fiber optic system with the suspension of oxygen-sensitive heterogeneous biocatalysts [48].

**Figure 6:**
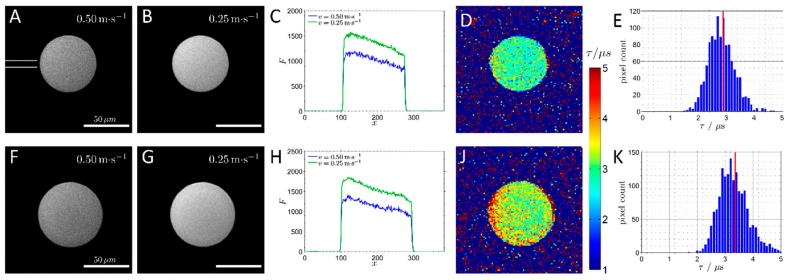
Luminescence lifetime imaging of agarose beads with immobilized Ru(dpp)_3_ immersed in air-saturated buffer (**A**–**E**) and in buffer with enzymatically depleted oxygen (**F**–**K**). The luminescence intensity of the beads is lower at higher scan speed (**A**,**B**,**F**,**G**). (**C**, **H**) The intensity profiles across the central part of the beads, as marked by two white lines in (**A)**. (**D**,**J**) The luminescence lifetime images. (**E**, **K**) Pixel lifetime distributions of the beads shown in (**D**) and (**J**) with the mean values marked by red lines. Reproduced with permission from reference [64]. Copyright (2016) American Chemical Society.

To obtain higher spatial resolution, it is necessary to use more sophisticated instrumentation (Figure 5) that enables the interfacing of the reaction system with cameras or microscopy lenses. Opto-chemical sensing in combination with confocal laser scanning microscopy (CLSM) has allowed the determination of internal parameters (e.g., pH) in a time and space-resolved manner. In the first examples, referenced fluorescence intensity measurements of the internal pH were accomplished. [50]. Intensity measurements in CLSM have been also used for the quantification of self-fluorescent molecules, with applications inside particles [39]. Fluorescence lifetime provides advanced measurement capabilities, eliminating signal distortion dependent on the scanning depth, which is a well-known problem of intensity-based measurements in CLSM [54]. Internal pH changes at spatial resolution have been monitored in hydrogels and PEG microparticles using fluorescence lifetime microscopy techniques. Unfortunately, lifetime and referenced measurements in CLSM depend on high-cost instrumentation that cannot be adapted to real-life reactor configurations and has limited throughput capacity. The application of multiphoton laser scanning microscopy [73,74] addresses some of the known limitations of measurements in CLSM. Multiphoton microscopy has been used to determine concentration gradients in hydrogel-encapsulated biocatalysts [73,74]. High spatial and temporal resolution is obtained, thus enabling multiple conversion events in immobilized biocatalysts to be monitored simultaneously. A practical solution for the use of intensity measurements in CLSM is based on intrinsically ratiometric dyes. This allows self-referenced measurements. For example, a recently presented methodology [67] is based on a pH-sensitive fluorescent protein. The superfolder yellow fluorescent protein (sYFP) [67,75,76] is as a powerful ratiometric pH indicator suitable for use both in solution and within porous materials [67,75,76]. sYFP belongs to a new generation of *Aequorea victoria* fluorescent proteins with improved stability and folding kinetics. The sYFP is highly stable and its pH-dependent fluorescence covers the relevant (neutral) pH range [67,75,76]. Optical pH sensing in solid materials based on sYFP is applicable to microscopic imaging analysis. In another example, fluorescence spectroscopy was applied to measure the acid microenvironment of a silica support by using a fluorescence labeled protein [77]. A recently described method allows for the measurement of luminescence lifetimes in conventional CLSM, based on monitoring the relaxation of the dye molecules to a new steady state upon the onset of excitation. This contrasts the more conventional time-domain methods for the determination of excited state lifetimes, wherein relaxation to the ground state is measured after the excitation is finished. The principle of operation is not limited to a common point-scanning CLSM but can be applied in much faster line-scanning microscopes also (Figure 6) [64].

## 3. The Application of Intraparticle Sensing for the Development of Immobilized Enzymes as Biocatalysts

It was already emphasized that the intraparticle microenvironment can affect the performance of immobilized enzymes. Substrate and product concentration of the microenvironment can differ from the corresponding concentrations in bulk liquid. Evidence of the enzyme’s microenvironment is an important basis for rational strategies for the development the immobilized enzyme showing the desired properties. The pH and O_2_ concentration are two particularly important variables of the microenvironment. Both have a prominent role in biocatalysis. Many enzymatic reactions release or take up protons. Moreover, O_2_ is a common substrate in many oxidation reactions. pH and O_2_ gradients can form between particle and bulk when reactions are catalyzed by immobilized enzymes. A specific challenge for O_2_ supply to enzymes immobilized on solid supports is the low solubility of O_2_ in water. An O_2_ transfer rate into the particle that is comparable to, or even lower than, the enzymatic reaction rate results in a dramatic drop in the intraparticle O_2_ concentration. This in turn affects the kinetics of the enzymatic reaction. In this scenario, O_2_-dependent heterogeneous biocatalysts offer a very low apparent activity. On the other hand, the pH gradients arise due to partition effects within the solid material or higher proton release/consumption rates compared to the physical proton transfer rate. As a consequence, internal pH gradients can influence the activity, stability and selectivity of the heterogeneous biocatalyst. In both cases, determining whether the apparent catalytic properties are due to immobilization effects or the intraparticle environment is an important requirement for targeted biocatalyst optimization.

Intraparticle measurements haven proven quite useful for the advanced characterization of immobilized enzymes [12,14,20,49,50,51,58,59,62,78,79,80]. They were important to optimize the solid-supported biocatalysts and the chemical transformations catalyzed by them. They provide an enriched set of data important to perform reaction modeling. Finally, they enable new strategies of reaction control. Table 1 summarizes the different approaches used for the determination of pH and O_2_ in solid supports for enzyme immobilization. The table describes the method of opto-chemical sensing used and it points out the overall system and the mode of data acquisition used. The systems used are discussed in some detail, and each method is commented on regarding the opportunities it provides. The spatio-temporal resolution of the method is also analyzed to distinguish the methods providing space-averaged data from the methods enabling imaging within the solid support. Table 1 highlights studies, in particular, that have applied characterization of the internal environment of an immobilized enzyme to process optimization. The advantages provided by internal measurements can be summarized as follows.

### 3.1. Identification and Quantification of Diffusion Limitations

The quantification of space-averaged intraparticle oxygen concentrations in porous polymethacrylate enzyme porous supports was accomplished by labeling a carrier with an O_2_ sensitive luminophore. The phase modulation technique was used for measurement. Formation of a large gradient between the O_2_ concentrations in bulk solvent and the internal environment of the carrier was detected, clearly indicating limitations in the supply of O_2_ co-substrate into the solid support [48]. Internal acidification of a particle’s microenvironment has been identified in proton-releasing reactions, showing the different working microenvironment inside the particle compared to the bulk solution [49,50,59,67].

### 3.2. Dissection of the Influence of Physical and Biochemical Factors on Catalytic Effectiveness

A clear distinction between physical and biochemical factors of the effectiveness of the immobilized enzyme is possible. The availability of internal concentrations allows for the establishment of a kinetic model in which transport effects are decoupled from the enzymatic reaction [66]. Following this procedure, the intrinsic loss of activity by immobilization can be identified [48,66].

### 3.3. Screening of Suitable Immobilization Procedures

Optimization of the carrier’s geometrical parameters is of high importance in the rational design of immobilized biocatalysts. Additionally, immobilization conditions can be optimized in a target-oriented manner [50,62].

### 3.4. Guidance for Biocatalytic Process Intensification

In the case that the effectiveness of the immobilized enzyme may be limited by diffusion, the measurements by internal sensing will reveal it. The main variables of the solid biocatalyst (particle parameters; enzyme loading) can be varied rationally to remove the transport limitation. Internal sensing is useful for verification of the effect. The approach was used for the enhancement of the effectiveness of immobilized oxidases [65].

### 3.5. The use of Reaction-Diffusion Modeling for Biocatalytic Process Development

The availability of internal concentration data strongly supports the use of (dynamic) modeling for the characterization and optimization of enzymatic reactions. The work of Zavrel and colleagues demonstrates explicitly that the estimation of kinetic and mass transfer parameters for enzyme immobilizates should not be based on external data alone [74]. In a recent study, Nidetzky and co-workers have applied dynamic modeling to time-resolved internal pH data recorded during the reaction of immobilized cephalosporin C amidase [79]. Their procedure yielded estimates for the effective proton diffusivity in different enzyme carriers (mass transfer parameter) and also revealed the intrinsic amidase activity in different carrier-bound preparations of the enzyme (biological parameter) [79]. The availability of those two parameters, which are not readily accessible from experiments alone and which both affect the overall enzyme effectiveness factor, is shown to be instrumental to the selection of a suitable carrier.

### 3.6. Biocatalytic Process Design

This was shown in work of Spieß and colleagues [49,50]. Recently, the changes in microenvironmental pH and in operational enzyme stability were investigated for the biocatalytic reaction of immobilized cephalosporin C acylase. The cephalosporin C acylase releases protons during the reaction. The enzyme was covalently bound on an epoxy-activated porous support. The microenvironmental pH change in the immobilized enzyme during the reaction was detected by labeling the enzyme with the pH-sensitive dye fluorescein. The high catalytic rate in the initial phase of conversion resulted in a sharp intraparticle pH gradient, which was likely the key factor of low operational stability. Accordingly, a novel strategy for a two-stage catalytic process was developed to reduce the reaction rate of stage I at a low temperature to preserve enzymatic activity and to shorten the duration of catalysis at a high reaction temperature in stage II [51]. Additionally, the buffer strength was settled based on intraparticle measurement for the design of stable continuous operation in a fixed-bed reactor [80].

### 3.7. New Strategies for Control of the Reaction

Innovative process control strategies based on the on-line monitoring of internal parameters can be established. Recently, the pH value a biphasic oxidoreduction system was controlled by using a novel DLR measurement-based control concept. The hydrophobic prochiral substrate acetophenone was reduced to 1-phenylethanol by the *Lactobacillus brevis* alcohol dehydrogenase, immobilized in a polyvinyl alcohol hydrogel matrix and suspended in an acetophenone/*n*-heptane solution. It was demonstrated that DLR-based pH control maintained a stable process pH for at least 105 h duration within a range of 0.2 pH-units [58].

### 3.8. Sensing in Microreactors

**Figure 7:**
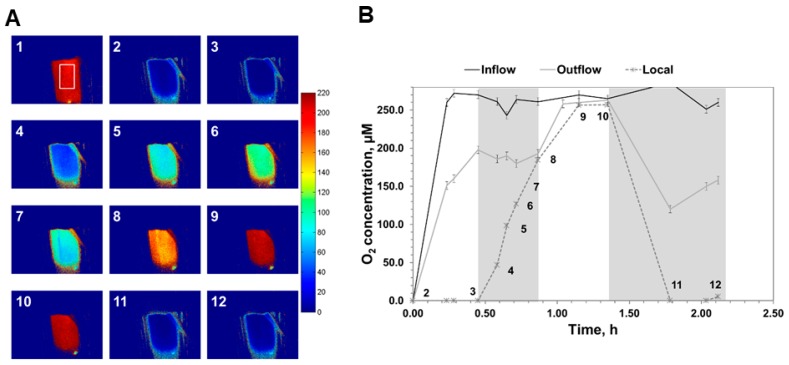
Results of internal and external O_2_ concentration measurements during the operation of a falling-film microreactor containing immobilized D-amino acid oxidase on the microchannel walls of the plate. Panel (**A**) shows the local O_2_ concentration on the plate in hPa (1 hPa is equivalent to 1.16 μM at 30 °C). The region of interest for measurement is comprised of a rectangle located at the lower end of the plate, as indicated in the image #1 of panel (**A**). Panel (**B)** shows O_2_ concentration measurements with the flow-through sensors at the reactor entrance and exit and compares these measurements to the results of on-plate O_2_ concentration measurements. Numbering is used to identify images from the upper panel that correspond to the local O_2_ concentrations shown in the lower panel. In the first phase of the experiment (#2–3), the FFMR was operated with nitrogen flow, resulting in a complete deoxygenation of the plate. When the air flow was switched on (#3–8), the local O_2_ concentration increased sharply, revealing a highly effective O_2_ supply from the gas phase to the liquid phase. Eventually, air saturation was reached despite consumption of O_2_ for the enzymatic reaction (#9–10). Deoxygenation was only possible when air was again replaced by N_2_ (#11–12). Figure was adapted from reference [83] with permission from John Wiley and Sons.

There is high interest in the use of micro-structured flow reactors for chemical synthesis [81]. In these miniaturized systems, gas or liquid is passed in single or multi-phase flow through small channels of typically several ten to hundred µm in size. Reactions may take place in the continuous phase or at the surface of the microchannel walls. Internal sensing along the microchannel wall(s), therefore, constitutes a highly promising element of process monitoring and control in microreactors [46,47,82]. For example, advanced non-invasive opto-chemical sensing is applied to measure liquid-phase oxygen concentrations in both in and out-flow, as well as directly in the microchannels (width: 600 µm; depth: 200 µm) of a falling film microreactor (Figure 7). Modular luminescence lifetime imaging was used to determine the dissolved O_2_ concentrations directly on the surface of the microchannels. The measurement principle has been reported before, but it has not been applied for in-line sensing in microreactors (Figure 7). Measurements of the O_2_ concentration directly in the channel showed that the liquid side mass transfer coefficient for O_2_ governed the overall gas/liquid/solid mass transfer and that the O_2_ transfer rate (≥ 0.75 mM·s^−1^) vastly exceeded the maximum enzymatic reaction rate in a wide range of conditions [83]. In another example, on-line sensing was established in a wall-coated enzyme microreactor. Phosphorescence lifetime was applied, not only to collect time-resolve single point measurements near the inlet and outlet, but for imaging of the oxygen concentration along the whole fluidic path. This allowed overcoming the well-known restrictions of using outlet-inlet data only. Thus, in addition to the spot measurements, the whole oxygen concentration distribution was imaged [84].

## 4. Spatiotemporal Sensing of Internal pH and Oxygen: An Update

**Figure 8:**
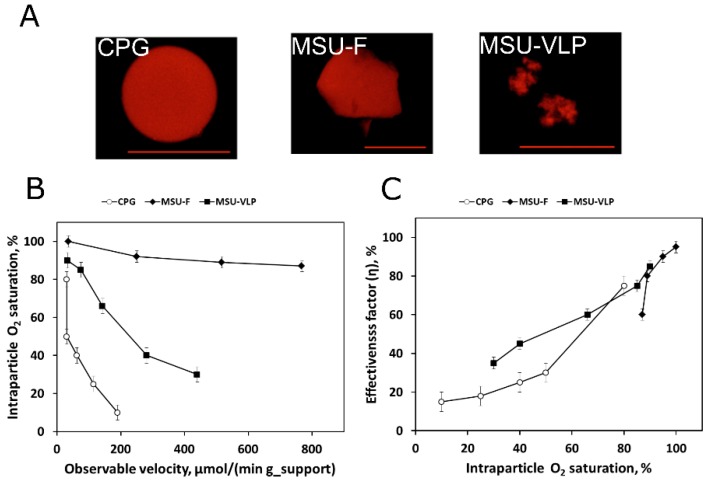
The effects of intraparticle oxygen concentration (expressed as relative air saturation in percent) on the activity of silica-based biocatalysts. (**A**) Confocal fluorescence images of the oxygen-sensitive luminescence dye immobilized on different porous silica supports (from left to right, CPG, MSU-VLP and MSU-F silica materials). (**B**) The dependence of the O_2_ concentration inside the porous support (at apparent steady state) on the velocity of D-methionine oxidation by the immobilized oxidase (D-amino acid oxidase) biocatalyst. (**C**) The dependence of the enzyme effectiveness of the biocatalyst on the intraparticle O_2_ concentration. All reactions were performed at 30 °C using air-saturated potassium phosphate buffer (50 mM; pH 8.0). Adapted with permission from reference [65]. Copyright (2015) American Chemical Society.

The quantification of spaced-averaged intraparticle O_2_-concentrations is now routinely applied to different porous supports containing immobilized oxidases [12,14]. Recent advances involve application of the method to new materials, as well as its use in the context of biocatalytic reaction intensification. Further method-developments target improvements in spatiotemporal resolution [64,65]. For example, the application of internal sensing enabled the optimization of geometrical properties of porous silica carriers for biocatalytic process intensification through enhanced mass transport (Figure 8). The development of luminophore-doped oxygen-sensing silica materials was connected with a modular strategy of enzyme immobilization. This shows the general applicability of the method for the design of an oxygen-dependent biocatalyst on a porous silica support. Mesostructured silica surpassed controlled pore glass by ≥10-fold in terms of immobilized enzyme effectiveness at high loading of oxidase activity. Using a detailed comparison of time-resolved O_2_ concentration profiles in solution and inside porous support, the effect was shown to result exclusively from variable degree of diffusion-caused limitation in the internal O_2_ availability [65].

Furthermore, internal measurements were instrumental in the development of a strategy for the enhanced supply of O_2_ into immobilized oxidases. The idea was to use co-immobilized catalase for the conversion of H_2_O_2_ and so release O_2_ inside the solid support at a concentration that would not be achievable by entraining O_2_ gas into the liquid bulk phase. Under oxygen hyper-saturation, the activity of the immobilized oxidase would be boosted due to a kinetic effect. Using optical sensing to measure the O_2_ concentration in the liquid, but also in the solid phase, it was shown that internal super-oxygenation of the support was made possible under these conditions, resulting in an inverted (that is, negative) O_2_ concentration gradient between the bulk and the particle. The internal O_2_ concentration exceeded, by up to 4-fold, the limit of atmospheric-pressure air saturation in solution. This strategy may find an application for the bubble-free oxygenation of O_2_-dependent enzymes co-immobilized with the catalase, whereby enhanced internal availability of O_2_ would contribute to biocatalytic reaction intensification [78].

**Figure 9:**
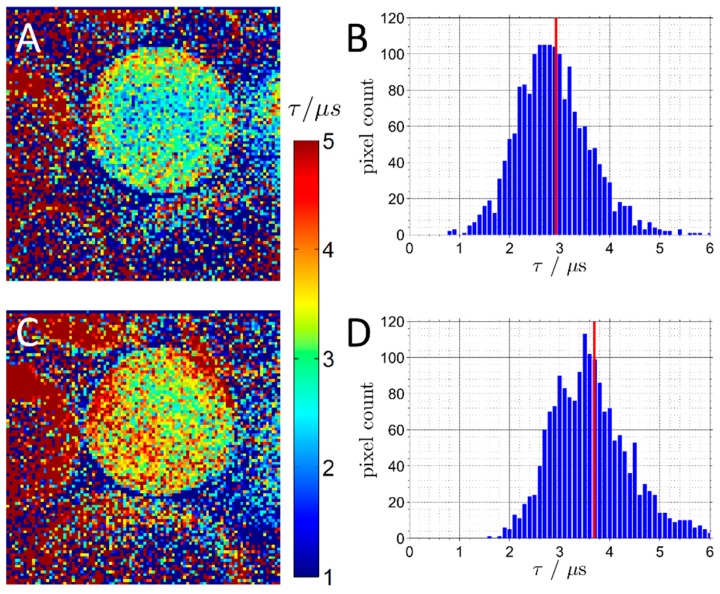
Luminescence lifetime images of beads with immobilized enzyme and Ru(dpp)_3_. Panels show imaging results under flow with (**C**,**D**) and without (**A**,**B**) substrate, resulting in different oxygen concentrations. (**B**,**D**) Pixel lifetime distributions of the beads are shown in (**A**) and (**C**), with the mean values marked by red lines. Adapted with permission from reference [64]. Copyright (2016) American Chemical Society.

Opto-chemical sensing in combination with microscopy has the potential to determine the internal pH and O_2_ concentration in real-time and with spatial resolution. Unfortunately, lifetime or referenced measurements in CLSM depend on high-cost instrumentation that cannot be adapted to real-life reactor configurations. Recently, as shown in Figure 9, a new method based on a variable excitation time determined by the scanning velocity was implemented in a CLSM for the resolution of internal O_2_ concentration [64]. The method allows phosphorescence lifetime imaging, thus spatiotemporal resolution within porous enzyme carriers. It has been applied for the study of oxygen depletion in particles containing immobilized lactate oxidase under packed-bed reactor configuration.

**Figure 10:**
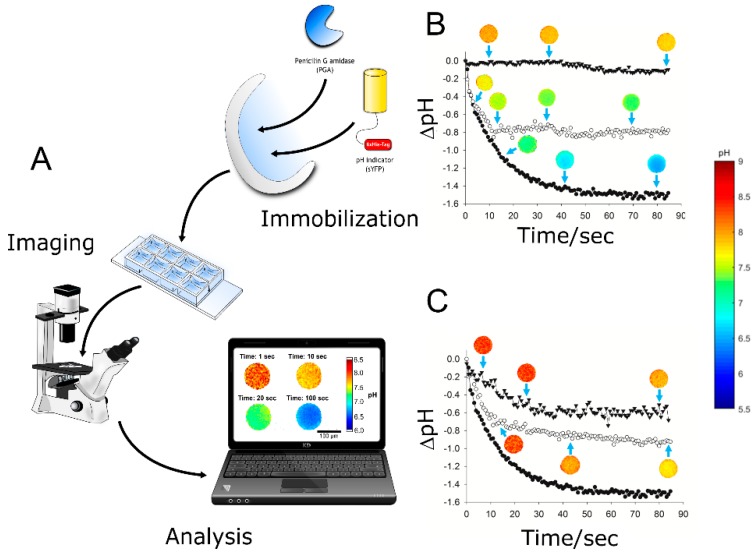
Measurement of intraparticle pH by sYFP-immobilized fluorescent protein. Panel (**A**) shows the set-up of the measurement. (**B**,**C**) show the evolution of ΔpH (internal pH – bulk pH) in reaction time courses of different enzyme catalysts. Panel (**B)** shows the effect of the penicillin acylase loading (per gram of carrier): 805 U (●), 130 U (○) and 27 U (▼). Panel (**C**) shows the effect of the sodium phosphate buffer concentration: 10 mM (●), 100 mM (○) and 200 mM (▼). The penicillin acylase loading used 805 U g^–1^ of carrier, and 20 mM penicillin G was used. Images of catalyst particles corresponding to each reaction are shown for different time points (blue arrows). Adapted with permission from reference [67]. Copyright (2018) American Chemical Society.

A new method for spatiotemporally resolved measurement of pH in immobilized enzymes has been implemented based on immobilized sYFP [67]. Its application was demonstrated for monitoring the internal pH of an immobilized penicillin acylase during the hydrolysis of penicillin. Conversion of the penicillin releases a proton. It was shown that sYFP is as a powerful pH indicator for ratiometric optical sensing that facilitates applications in real time within porous materials. A reaction setup was used in which agarose particles containing immobilized sYFP and penicillin acylase (catalyst particle) were kept static in stagnant suspension. The experiment was designed to monitor the initial reaction rate, immediately after addition of the substrate (Figure 10). The time course consists of a pronounced pH drop within the first seconds of reaction, followed by a slower phase of pH decrease. At apparent steady state, a pH difference of ~1.5 pH units between bulk and the catalyst particles prevailed. Both the dynamics and magnitude of the pH gradient reflect the coupling of proton-releasing reaction confined to the solid surface of the catalyst particle’s pores and the molecular diffusion events inside these pores.

## 5. Conclusions

Summarizing, the main points of this review article are the following. (1) Characterization of the internal liquid milieu in which solid-supported immobilized enzymes operate is crucial to understand the behavior of the enzymes in terms of activity and stability. That understanding is important to assess competing immobilization methods and to develop rational strategies for the optimization of performance of immobilized enzymes. (2) Opto-chemical sensing is a powerful method for the characterization of the internal liquid milieu. It is well developed for measuring the pH and the O_2_ concentration. However, a range of other analytes are within its scope. Opto-chemical sensing can be applied in a versatile manner regarding the enzyme, the solid material, the analytical set-up and read-out system, and the integration into the reaction/process configuration. Opportunities and current limitations of opto-chemical sensing in the context of immobilized enzymes are discussed. The claims made in this review are supported by original results from recent studies from different laboratories, including the ones of the authors. Important areas of future development include the following. Broadening of the analyte scope of the analytical techniques as the field of opto-chemical sensings makes progress (NH_4_, CO_2_, temperature, etc.); the application of new support materials for enzyme immobilization (metal and covalent organic frameworks, electrospun nanofibers, ceramic structured membranes, etc.); improved catalyst characterization for deepened mechanistic understanding; and generating synergy between advanced catalyst characterization and improved operational use of the catalyst, using defined reactor configurations under in operando conditions.

## Figures and Tables

**Table 1:** Internal opto-chemical sensing in heterogeneous biocatalysts.

Analyte	Methodology	System	Spatiotemporal Resolution	Relevance and Comments	Ref.
pH	Luminescence intensity measurements Fiber optic connected to spectrofluorometer	Porous support containing labeled enzyme: penicillin amidase Stirred tank reactor Fixed bed reactor	Real-time monitoring Space-averaged data	Method development Identification of pH gradients: 1.5-3 units Modeling performed and validated Low signal-to-noise ratio can complicate analysis of stirred suspensions	[49]
pH	Luminescence intensity-based measurements Dual wavelength rationing; CLSM	Porous support containing labeled enzyme: penicillin amidase -FITC (pH indicator), FITC coupled to immobilized enzyme Fixed-bed reactor	No real-time monitoring Spatial resolution	Biocatalyst screening: pH influence on the selectivity of kinetically controlled reactions. Study of enzyme loading, particle and pore size, surface modification, and carrier selection	[50]
pH	Fluorescence ratiometric imaging CSLM	Polymeric membrane containing pH-sensitive nano-hydrogels and glucose oxidase FITC (pH indicator) T-Red (reference dye)	Real-time monitoring Spatial resolution	pH profile inside the membrane determined in buffer of different pH or at different glucose concentrations, affecting the pH due to reaction of glucose oxidase Internal pH decreased with the increase in glucose concentration, incubation time, and diffusion distance	[55]
pH	DLR using phase modulation; fiber optic system	Fluorescent labeled porous support containing immobilized enzyme Stirred particle suspension	Real-time monitoring Space-averaged data	Method development Biocatalyst design: pH gradient depends on geometrical features of the carrier Correlation between steady-state kinetic analysis of immobilized enzyme and intraparticle elucidation Internal pH monitoring pH gradient between bulk and particle (biocatalyst design) Correlation between steady-state kinetic analysis of immobilized enzyme and intraparticle elucidation	[59,62]
O_2_	Phase modulation technique; fiber optic system	Phosphorescent labeled porous carriers containing immobilized glucose oxidase Stirred particle suspension	Real-time monitoring Space-averaged data	Method development Compatible with different carrier surface modifications, dyes adsorbed directly in the carrier matrix Oxygen gradient depends on immobilization approach and informs about intrinsic immobilization chemistry	[48]
O_2_	Phase modulation technique, fiber optic system	Phosphorescent labeled porous carriers containing immobilized D-amino acid oxidases Stirred particle suspension	Real-time monitoring Space-averaged data	Dyes adsorbed directly in the carrier matrix Oxygen gradient depends on immobilization approach and informs about intrinsic immobilization chemistry	[66]
3,5-Dimethoxybenzaldehyde-)	Two-photon laser scanning microscopy	Hydrogel beads suspended in an organic solvent containing immobilized benzaldehyde lyase	Real-time monitoring Spatial resolution	Method development Determination of intrinsic reaction parameters and mass transfer parameters Mechanism-based kinetic model in good agreement with experimental data	[73,74]
O_2_	Phase modulation technique, fiber optic system	Phosphorescent labeled porous carriers (silica based) containing immobilized D-amino acid oxidase Stirred particle suspension	Real-time monitoring Space-averaged data	Method development Biocatalytic process intensification through enhanced O_2_ transport	[65]
O_2_	Phase modulation technique, fiber optic system	Phosphorescent labeled porous carriers (polymethacrylate based) containing immobilized catalase Stirred particle suspension	Real-time monitoring Space-averaged data	Observation of the release of internal oxygen from H_2_O_2_ using immobilized catalase O_2_ hyper-saturation into the porous material	[78]
pH	DLR using phase modulation; fiber optic system	Polyvinyl alcohol (PVA) beads containing immobilized enzyme and phosphorescent labeled nanoparticles Stirred particle suspension	Real-time monitoring Space-averaged data	Method development Control strategy based on intraparticle pH	[58]
pH	Luminescence intensity measurements Fiber optic system	Porous support containing labelled enzyme (Cephalosporin C acylase) Stirred particle suspension	Real-time monitoring Space-averaged data	Process design assisted by intraparticle measurements Operational stability of the enzyme was increased by avoiding high internal acidification	[51]
pH	Luminescence spectroscopy	Porous support containing fluorescent labeled proteins	No real-time monitoring Spectral data	Method development Measurement of the acid microenvironment cause by material support	[77]
pH	DLR using phase modulation; fiber optic	Fluorescent labeled porous particles containing immobilized enzyme Stirred particle suspension	Real-time monitoring Space-averaged data	Modeling and simulation used to characterize the influence of geometrical features of the carrier Calculation of intrinsic parameters Prediction of immobilized enzyme effectiveness factors	[79]
O_2_	Luminescence lifetime in a CLSM	Porous agarose labeled with Ru containing immobilized lactose oxidase Fixed bed	No real-time monitoring Spatial resolution	Method development Implementation of simple lifetime measurement and phosphorescence lifetime imaging in a confocal laser scanning microscope (CLSM),	[64]
pH	Luminescence intensity measurements Fiber optic	Porous carrier containing labelled enzyme (Cephalosporin C acylase) Stirred particle suspension Fixed bed	Real-time monitoring Space-averaged data	Process design assisted by intraparticle measurements. Selection of buffer was assisted by measurement of intraparticle environment	[80]
pH	Fluorescence ratiometric measurements in CSLM	Fluorescent labeled porous carrier containing immobilized enzyme Fixed bed	Real-time monitoring Spatial resolution	Method development. Identification of pH gradients: 1.5-3 units Fluorescent labeling of diverse carrier materials by using sYFP Effects of catalyst loading and buffer strength on pH gradient	[67]
